# Supplementary figures and images for: Propionyl-CoA carboxylase subunit B regulates anti-tumor T cells in a pancreatic cancer mouse model
Source: eLife. 2025 Mar 11;13:RP96925. doi: 10.7554/eLife.96925 (PMC11896608; doi:10.7554/eLife.96925)

KPC aKO p-aKO

HSP90

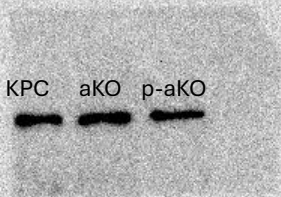

Supplement: Figure 3—source data 1. — Numbers in front of each file indicate if they are from the same gel. [file elife-96925-fig3-data1.zip › Figure 3-Source Data 1. Labeled Western Blot files/WB#4_HSP90.pdf]

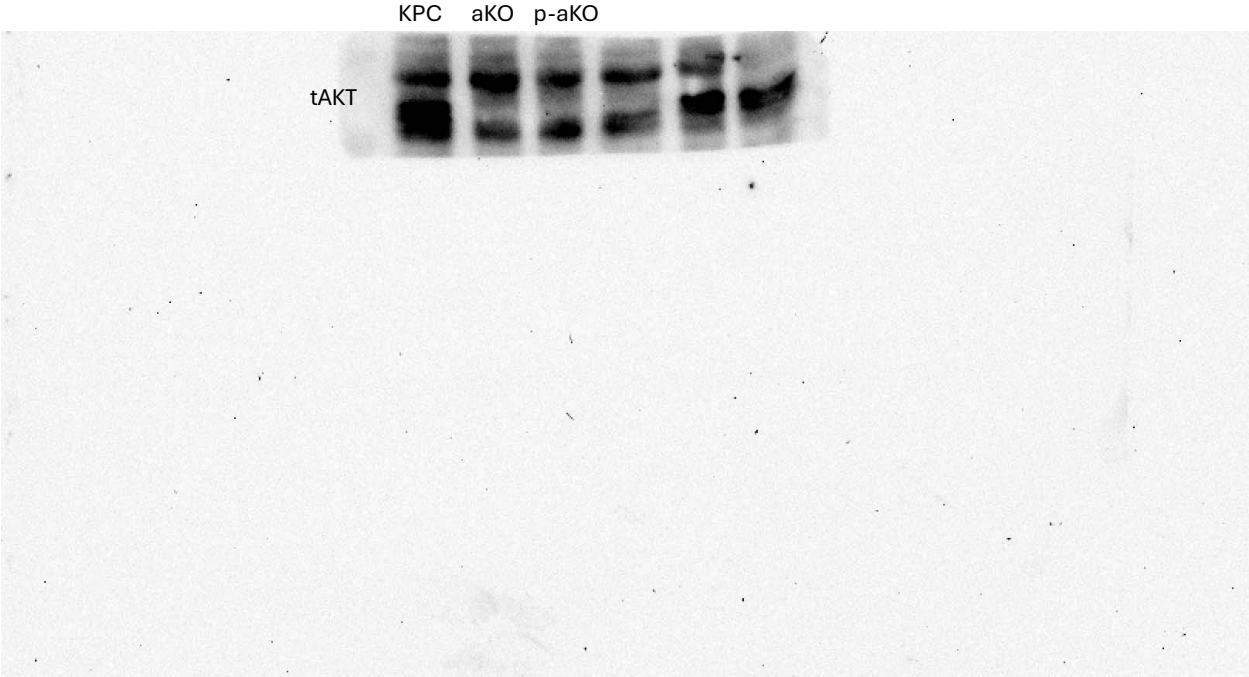

Supplement: Figure 3—source data 1. — Numbers in front of each file indicate if they are from the same gel. [file elife-96925-fig3-data1.zip › Figure 3-Source Data 1. Labeled Western Blot files/WB#2_tAKT.pdf]

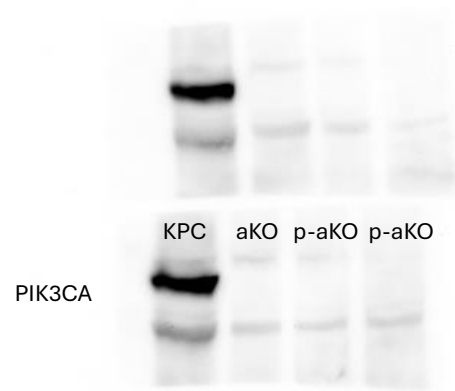

Supplement: Figure 3—source data 1. — Numbers in front of each file indicate if they are from the same gel. [file elife-96925-fig3-data1.zip › Figure 3-Source Data 1. Labeled Western Blot files/WB#1_PIK3CA.pdf]

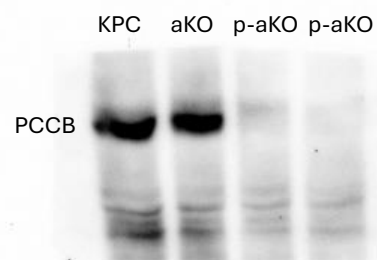

Supplement: Figure 3—source data 1. — Numbers in front of each file indicate if they are from the same gel. [file elife-96925-fig3-data1.zip › Figure 3-Source Data 1. Labeled Western Blot files/WB#1_PCCB.pdf]

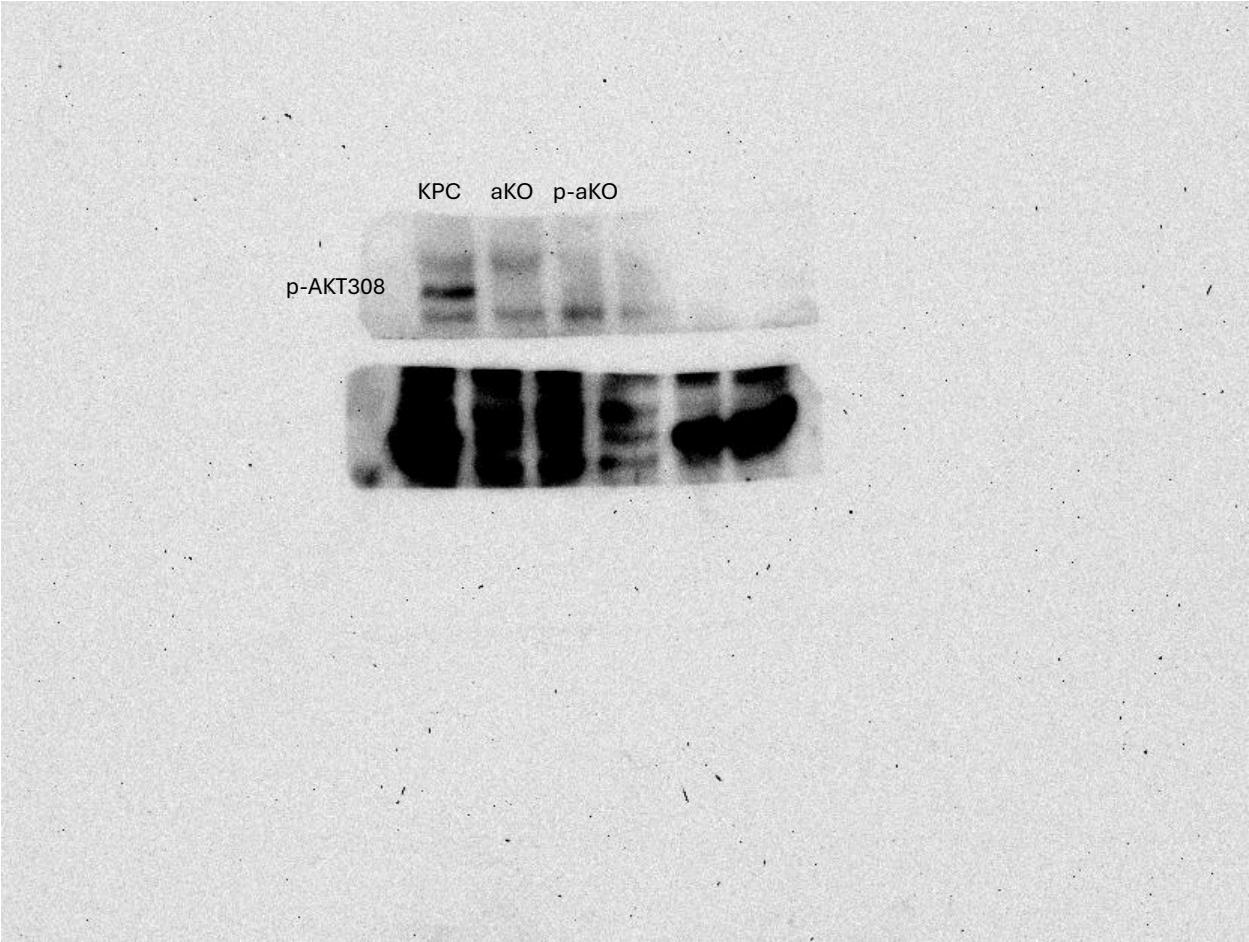

Supplement: Figure 3—source data 1. — Numbers in front of each file indicate if they are from the same gel. [file elife-96925-fig3-data1.zip › Figure 3-Source Data 1. Labeled Western Blot files/WB#2_pAKT308.pdf]

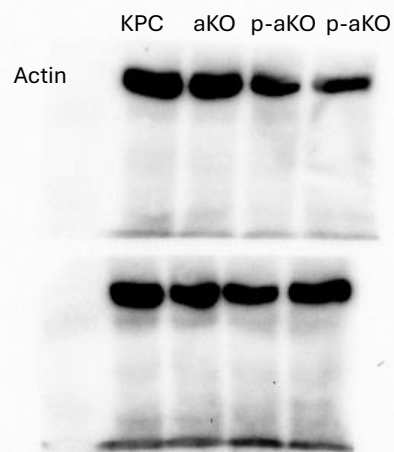

Supplement: Figure 3—source data 1. — Numbers in front of each file indicate if they are from the same gel. [file elife-96925-fig3-data1.zip › Figure 3-Source Data 1. Labeled Western Blot files/WB#3_Actin.pdf]

pAKT473

KPC aKO p-aKO

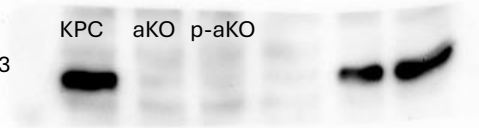

Supplement: Figure 3—source data 1. — Numbers in front of each file indicate if they are from the same gel. [file elife-96925-fig3-data1.zip › Figure 3-Source Data 1. Labeled Western Blot files/WB#2_pAKT473.pdf]

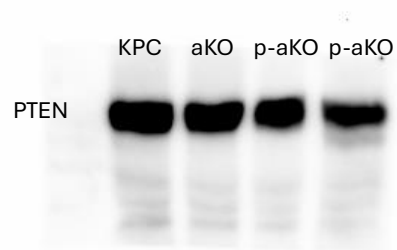

Supplement: Figure 3—source data 1. — Numbers in front of each file indicate if they are from the same gel. [file elife-96925-fig3-data1.zip › Figure 3-Source Data 1. Labeled Western Blot files/WB#3_PTEN.pdf]

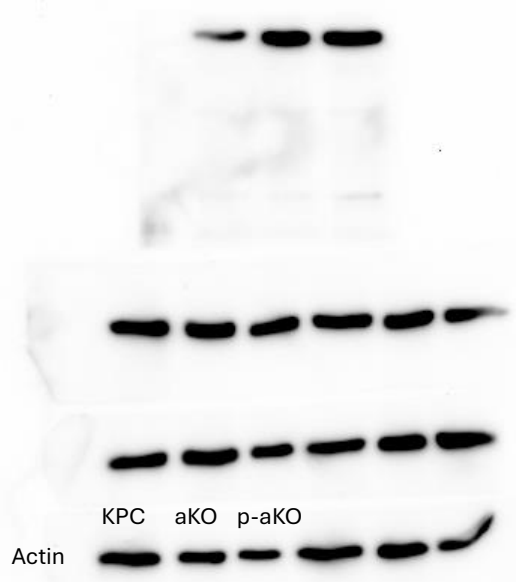

Supplement: Figure 3—source data 1. — Numbers in front of each file indicate if they are from the same gel. [file elife-96925-fig3-data1.zip › Figure 3-Source Data 1. Labeled Western Blot files/WB#2_Actin.pdf]

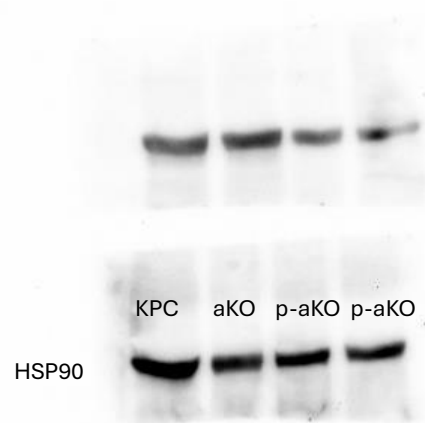

Supplement: Figure 3—source data 1. — Numbers in front of each file indicate if they are from the same gel. [file elife-96925-fig3-data1.zip › Figure 3-Source Data 1. Labeled Western Blot files/WB#1_HSP90.pdf]

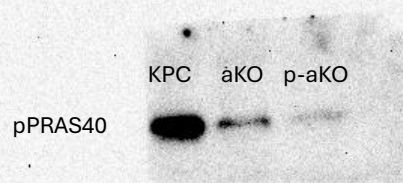

Supplement: Figure 3—source data 1. — Numbers in front of each file indicate if they are from the same gel. [file elife-96925-fig3-data1.zip › Figure 3-Source Data 1. Labeled Western Blot files/WB#4_pPRAS40.pdf]

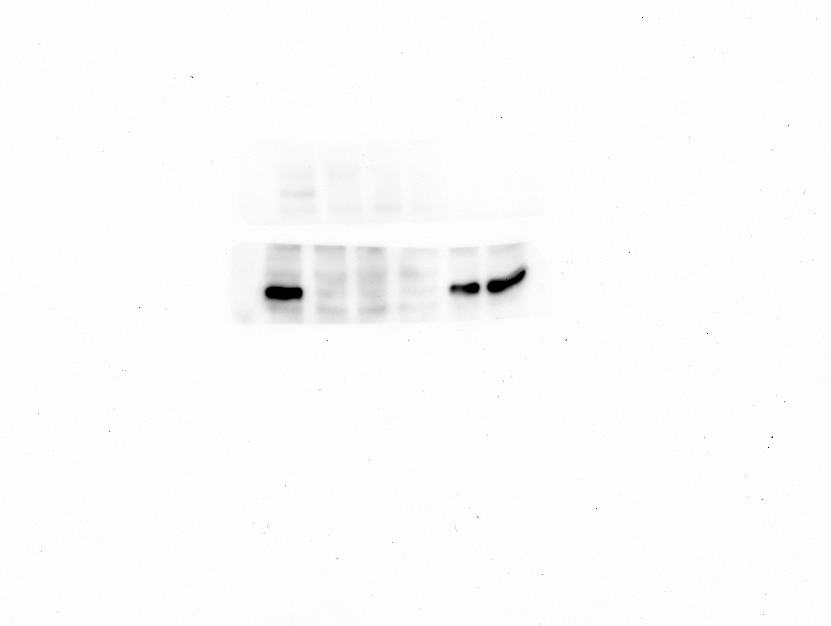

Supplement: Figure 3—source data 1. — Numbers in front of each file indicate if they are from the same gel. [file elife-96925-fig3-data1.zip › Figure 3-Source Data 1. Western blot files/3-pAkt473(bottom).png]

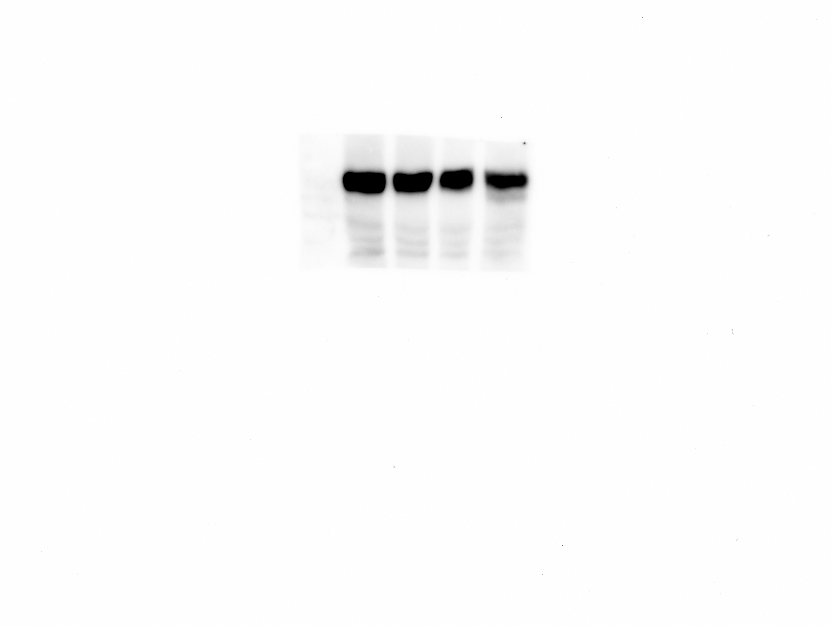

Supplement: Figure 3—source data 1. — Numbers in front of each file indicate if they are from the same gel. [file elife-96925-fig3-data1.zip › Figure 3-Source Data 1. Western blot files/2-PTEN.png]

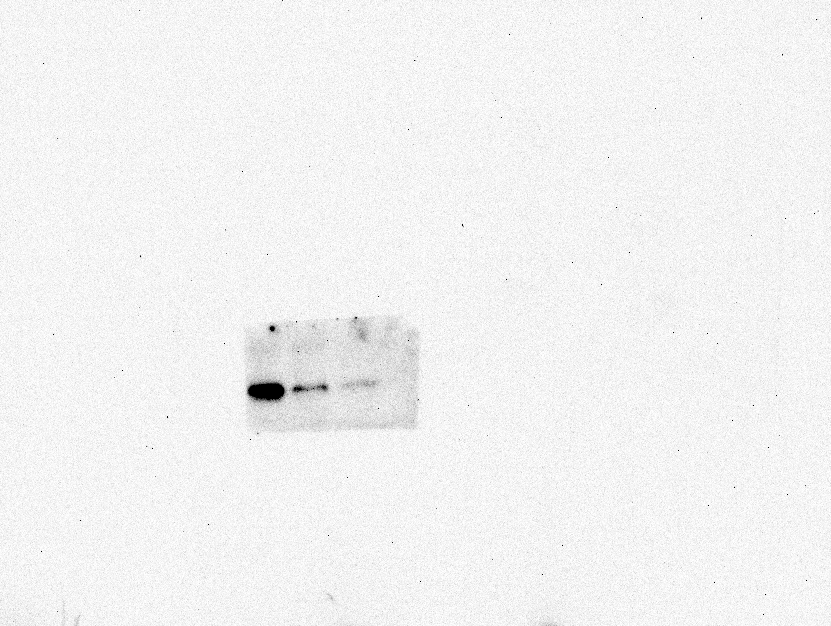

Supplement: Figure 3—source data 1. — Numbers in front of each file indicate if they are from the same gel. [file elife-96925-fig3-data1.zip › Figure 3-Source Data 1. Western blot files/4-pPRAS40.png]

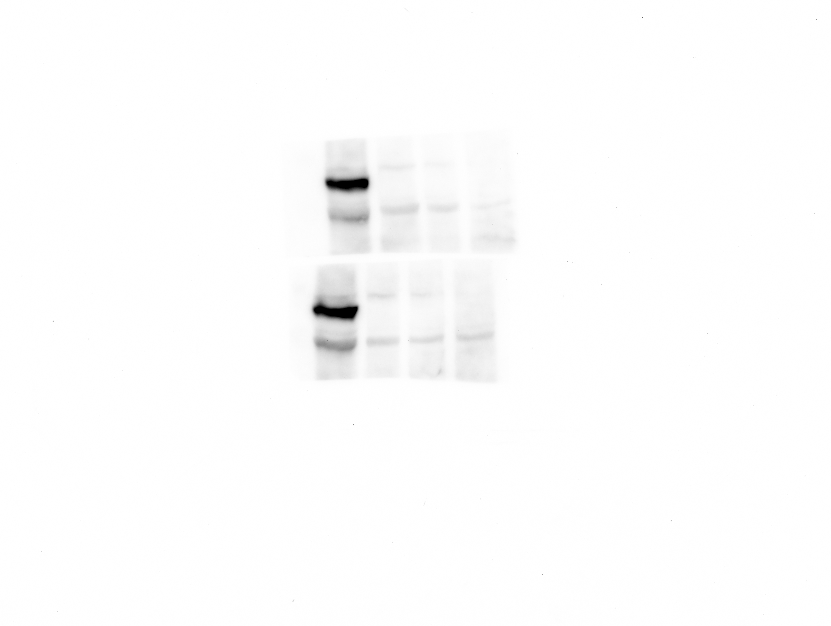

Supplement: Figure 3—source data 1. — Numbers in front of each file indicate if they are from the same gel. [file elife-96925-fig3-data1.zip › Figure 3-Source Data 1. Western blot files/1-PIK3CA.png]

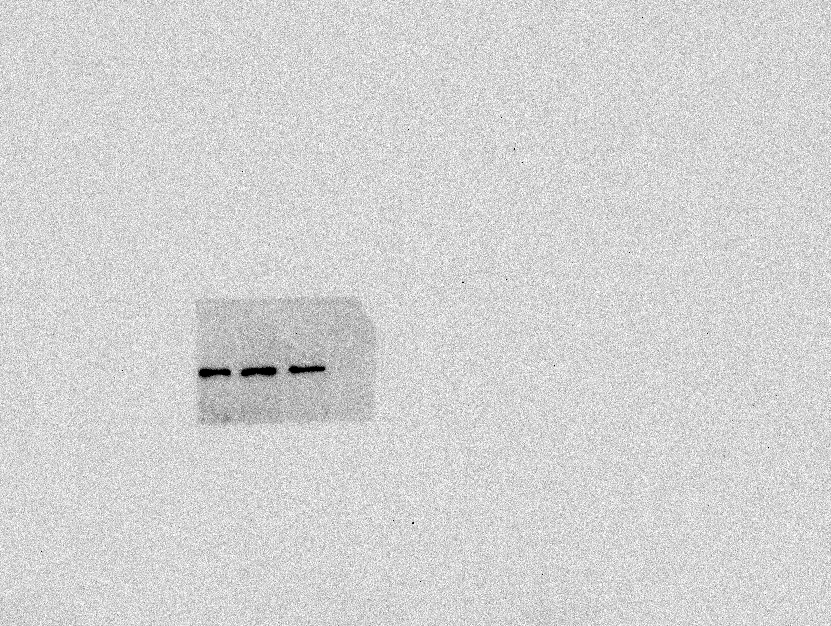

Supplement: Figure 3—source data 1. — Numbers in front of each file indicate if they are from the same gel. [file elife-96925-fig3-data1.zip › Figure 3-Source Data 1. Western blot files/4-HSP90.png]

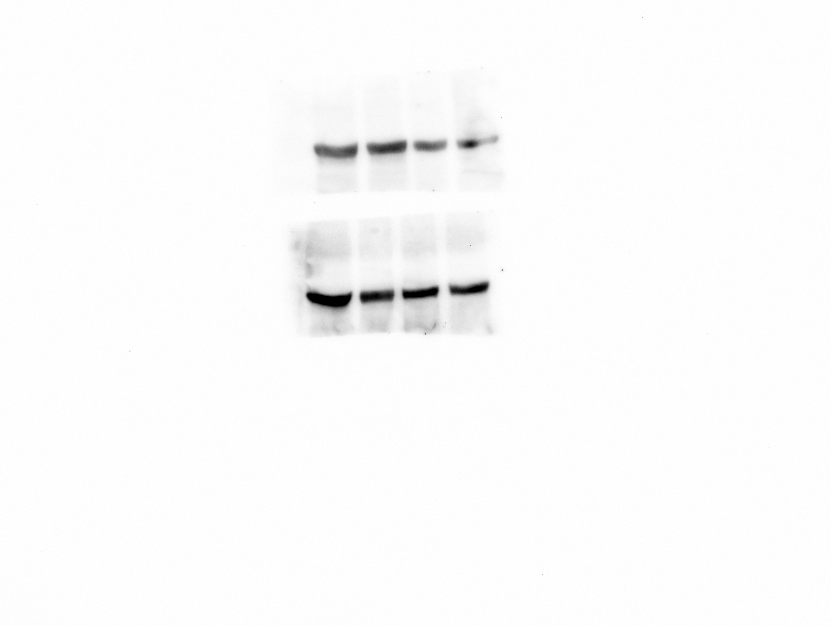

Supplement: Figure 3—source data 1. — Numbers in front of each file indicate if they are from the same gel. [file elife-96925-fig3-data1.zip › Figure 3-Source Data 1. Western blot files/1-HSP90.png]

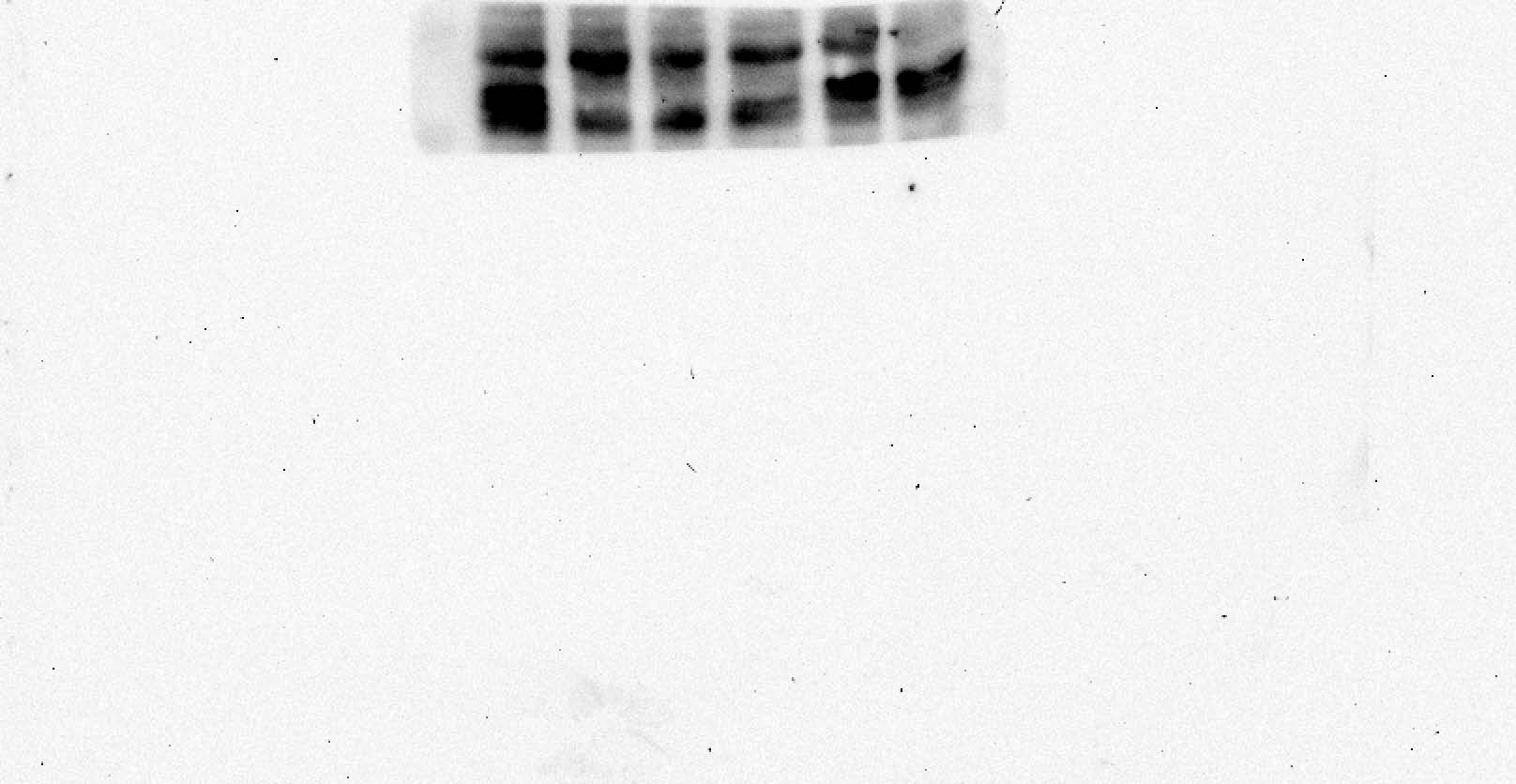

Supplement: Figure 3—source data 1. — Numbers in front of each file indicate if they are from the same gel. [file elife-96925-fig3-data1.zip › Figure 3-Source Data 1. Western blot files/3-tAKT.jpg]

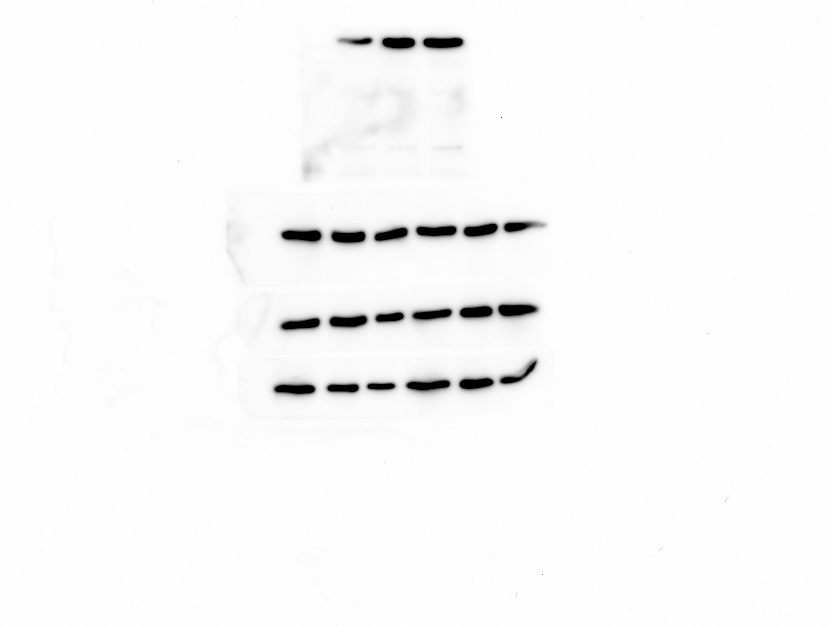

Supplement: Figure 3—source data 1. — Numbers in front of each file indicate if they are from the same gel. [file elife-96925-fig3-data1.zip › Figure 3-Source Data 1. Western blot files/2-Actin.png]

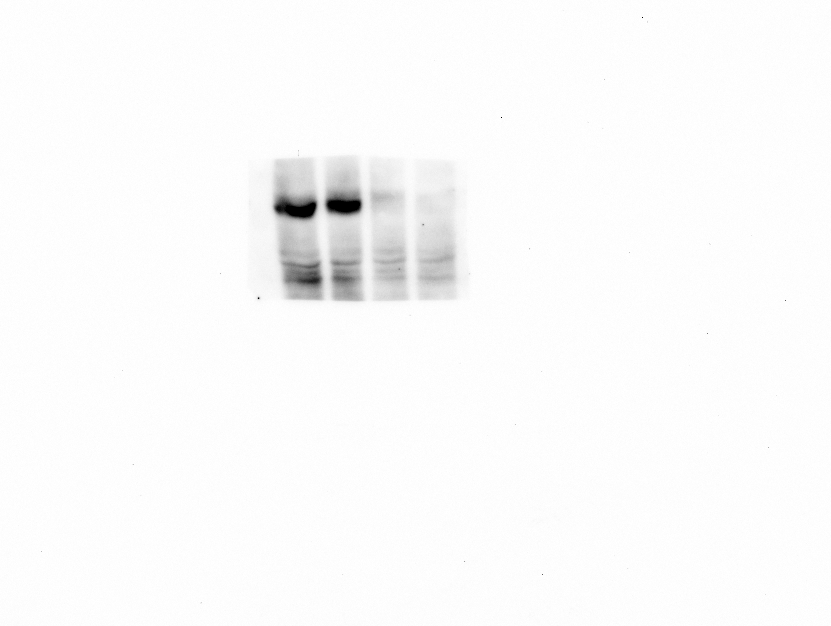

Supplement: Figure 3—source data 1. — Numbers in front of each file indicate if they are from the same gel. [file elife-96925-fig3-data1.zip › Figure 3-Source Data 1. Western blot files/1-PCCB.png]

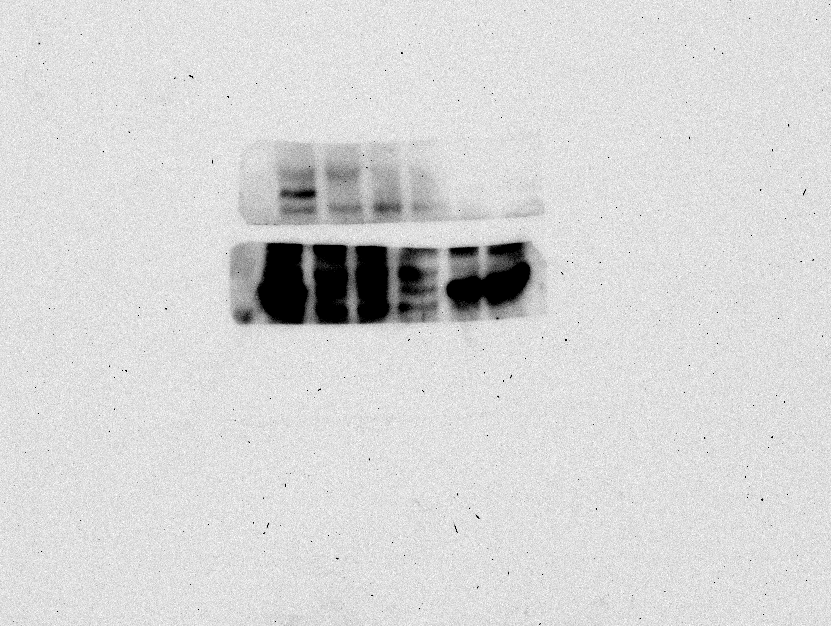

Supplement: Figure 3—source data 1. — Numbers in front of each file indicate if they are from the same gel. [file elife-96925-fig3-data1.zip › Figure 3-Source Data 1. Western blot files/2-pAkt308(top).png]

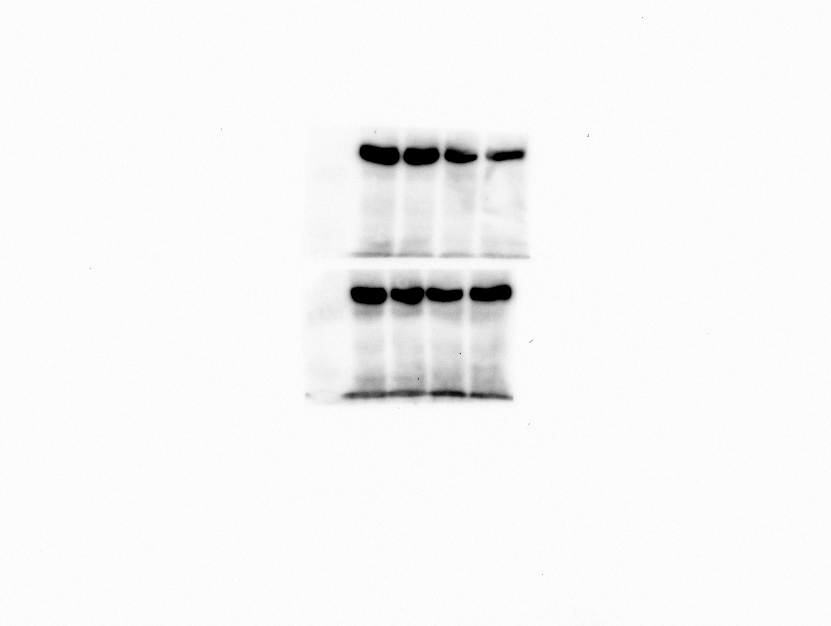

Supplement: Figure 3—source data 1. — Numbers in front of each file indicate if they are from the same gel. [file elife-96925-fig3-data1.zip › Figure 3-Source Data 1. Western blot files/3-Actin.png]
